# The impact of credit accessibility and information communication technology on the income of small-scale sugarcane farmers in Ndwedwe Local Municipality, KwaZulu-Natal Province, South Africa

**DOI:** 10.3389/fsufs.2024.1392647

**Published:** 2024-05-13

**Authors:** Nkosingiphile Samuel Zulu, Simphiwe Innocentia Hlatshwayo, Temitope Oluwaseun Ojo, Rob Slotow, Thobani Cele, Mjabuliseni Simon Cloapas Ngidi

**Affiliations:** 1 School of Agricultural, Earth and Environmental Sciences, College of Agriculture, Engineering and Science, University of KwaZulu-Natal, Pietermaritzburg, South Africa; 2 Functional Biodiversity, Centre for Functional Biodiversity, School of Life Sciences, College of Agriculture, Engineering and Science, University of KwaZulu-Natal, Pietermaritzburg, South Africa; 3 Department of Agricultural Economics, Obafemi Awolowo University, Ile-Ife, Nigeria; 4 Department of Plant, Food and Environmental Sciences, Faculty of Agriculture, Dalhousie University, Truro, NS, Canada; 5 Disaster Management Training and Education Center for Africa, University of the Free State, Bloemfontein, South Africa

**Keywords:** small-scale farmers, access to credit, ICT adoption, income, sugarcane, recursive bivariate probit

## Abstract

**Introduction:**

Access to credit and information and communication technology (ICT) plays a pivotal role in enhancing the practices of small-scale sugarcane farmers, impacting their financial, social, and economic wellbeing. However, many small-scale farmers need help accessing these resources, thereby affecting their ability to generate sustainable income. This study aimed to assess the factors influencing the adoption of ICT and access to credit and their subsequent impact on small-scale farmers’ income.

**Methods:**

Employing a multistage sampling technique, 300 small-scale farmers were selected as participants in the study. The recursive bivariate probit regression model was used to assess the factors affecting adoption ICT and a selectivity-corrected ordinary least square regression model was utilized to estimate the synergistic effect of ICT adoption and access to credit on the income of small-scale sugarcane farmers.

**Results and discussion:**

The findings revealed that approximately 77% of small-scale farmers had access to credit, while more than 80% had adopted ICT. The results derived from the recursive bivariate probit (RBP) regression model indicated that access to credit, education, and extension support positively and significantly influenced the adoption of ICT. Conversely, marital status and non-farm income exhibited a negative and significant influence on the adoption of ICT. Gender and marital status were positively and significantly associated with access to credit, whereas age, education, and non-farm income showed a negative and significant relationship on access to credit. Subsequently, a selectivity-corrected ordinary least square regression model analysis revealed that factors such as gender, marital status, extension, government support, and transportation costs positively and significantly influenced farmer’s income. In contrast, education, employment status, and non-farm income exhibited a negative and significant influence on income.

**Conclusion and recommendations:**

The study concludes that socio-demographic factors, such as gender, marital status, extension support, government support, and transportation costs, positively contribute to farmers’ income. Small-scale sugarcane farmer involvement in other non-farm activities is associated with reduced farm income. This implies that farmers’ livelihoods options are reduced as they can only focus on sugarcane development as a source of income. There is a pressing need to educate small-scale farmers on ICT and provide them with access to agricultural credit. Additionally, extension workers should offer advisory support to small-scale farmers requiring assistance in accessing agricultural credit. There is a need to train sugarcane farmers on different agricultural income generating activities to reduce their over-reliance on sugarcane development. By addressing the identified socio-demographic factors and implementing targeted policy interventions, stakeholders can foster an enabling environment for small-scale farmers to thrive, ultimately contributing to the sustainable development of the sugarcane sector and the broader agricultural landscape in South Africa.

## 1 Introduction

The population of South Africa predominantly relies on agriculture as its primary source of food and income, with ~70% of rural households depending on it for employment (Poole, 2017). Furthermore, agriculture serves as a crucial supplier of raw materials for the industrial sector, which, in turn, supports the market for manufactured goods and accounts for 10% of South Africa’s foreign exchange earnings (International Trade Administration, 2023). South African agriculture is characterized by a significant number of small-scale farmers who produce a variety of cash crops and food crops, including maize, beans, and vegetables, primarily for subsistence (Mkuhlani et al., 2020). Among these crops, sugarcane stands out as a vital cash crop, primarily cultivated by small-scale farmers to generate income. Sugarcane crop holds global significance due to its widespread use in everyday life and its industrial applications, which contribute to dietary and economic sustainability [South Africa Sugar Association (SASA), 2012]. The sugarcane sector plays a crucial role in South Africa’s economy by significantly contributing to the country’s GDP through various socioeconomic development initiatives. These initiatives focus on job creation, resource organization, income generation, and the development of transport and communication networks (Aliyu et al., 2018). Moreover, the sector generates an estimated R6 billion in direct annual revenue from exports to the rest of the world and the regional block of the Southern African Customs Union (SACU) (Thibane et al., 2023). However, despite its substantial contributions, the sugarcane sector faces significant capital and financing challenges. Small-scale sugarcane farmers in South Africa struggle to adopt Information Communication Technology (ICT), which hampers their access to essential information regarding credit opportunities. Consequently, this affects their ability to finance their production and generate income.

Access to credit and Information Communication Technology (ICT) are crucial resources for enhancing the practices of small-scale sugarcane farmers. Asongu et al. (2019) has emphasized that ICT offers essential tools that facilitate both lenders and borrowers in better understanding market risks, credit terms and conditions, and enhances their ability to manage information asymmetry and moral hazards. Siyao (2012) has highlighted that access to information technology is a significant resource for socio-economic development, enabling households to make informed decisions and improve their livelihoods. Olorunda and Oyelude (2008) has similarly asserted that access to information is vital for effective planning, decision-making, and program execution. In the modern era, agriculture increasingly relies on technological advancements, underscoring the importance of farmers having access to technical and scientific information to make informed decisions (Salehi et al., 2021). Furthermore, ICT has the potential to enhance the competitiveness of sugarcane farmers within the agricultural supply chain (Shikuku, 2019). Therefore, it is evident that sugarcane farmers with access to proper ICT are better positioned to access formal credit, and vice versa.

Access to credit is a condition wherein small-scale farmers can access specific sources of capital, enabling them to acquire essential inputs, such as improved seed cultivars, fertilizers, and machinery (Motsoari et al., 2015). It represents financial assistance that empowers small-scale farmers to fulfill the financial requirements for primary agricultural inputs essential for production (Abdallah, 2016; Adjognon et al., 2017; Afrin et al., 2017). According to Manganhele (2010) experiences in numerous developing communities have demonstrated that access to credit can accelerate the adoption of new technologies. Various studies have revealed that many farmers in developing countries, including South Africa, face resource limitations and liquidity constraints when purchasing necessary inputs, underscoring the critical importance of access to credit for them (Saqib et al., 2016, 2018; Chandio et al., 2018). Moreover, access to credit significantly contributes to the growth and development of the rural economy. Therefore, secure, and timely access to formal credit can facilitate a transition from subsistence farming to commercial agriculture, fostering a productive change (Saqib et al., 2018).

Despite the significant contribution that the adoption of ICT has on the ability of small-scale farmers to access credit, small-scale farmers are faced with difficulties in accessing and adopt ICT and credit which affects their ability to generate sustainable income. Most of the sugarcane small-scale farmers in South Africa are faced with severe challenges that include technical, economic, and social issues (Thibane et al., 2023). The policies or terms and conditions included in formal credit are difficult for farmers to adhere to. For instance, formal credit requires small-scale farmers to have reliable collateral while most farmers do not own land and others own very small sizes of land (1.5 hectares) (Salehi et al., 2021). The information included in formal credit is sometimes too complex for farmers to understand and requires a lot of administration. Chandio et al. (2021) stated that in most cases formal credit policies fail to address the small-scale farmers’ needs for a loan, also, they are not compatible with farm and personal objectives.

With regard to information technology, small-scale farmers do not adopt adequate information. Shanthy (2011) outlined that ICT typically involves a “top-down” approach where people at the top can be researchers, government, or policy makers who provide information with innovations that are sophisticated and provide them with extension services to deliver to farmers without any training. Therefore, farmers become reluctant and rely more on their traditional knowledge and informal lenders which are not adequate to sustain the production processes.

Improving access to credit and adopting ICT will enhance the agricultural production of small-scale farmers, thereby affecting farm income. Therefore, it is crucial to comprehend all the factors that impact small-scale farmers’ ability to obtain ICT and formal credit. This understanding will enable the development of interventions that enhance farmers’ capabilities and improve their agricultural performance. Several studies (Kuwornu et al., 2012; Chandio et al., 2016, 2021; Saqib et al., 2016) have explored the influence of access to credit on farmers’ production, while others have focused on the role of access to information in farmers’ production (Shanthy, 2011; Siyao, 2012; Salehi et al., 2021). However, there is a dearth of empirical research examining the combined effect of ICT and access to credit on sugarcane small-scale farmers’ income.

Despite existing literature exploring the individual effects of credit accessibility and Information Communication Technology (ICT) adoption on agricultural production, there remains a notable research gap regarding their combined impact on the income of small-scale sugarcane farmers in the Ndwedwe Local Municipality, KwaZulu-Natal Province, South Africa. While studies have examined the influence of access to credit on farmers’ production and income (Kuwornu et al., 2012; Saqib et al., 2016), and others have focused on the role of ICT in enhancing agricultural practices (Shanthy, 2011; Siyao, 2012), there is limited empirical research that considers the simultaneous effect of both factors on sugarcane farming income in this specific geographical context. Furthermore, existing studies primarily focus on either credit accessibility or ICT adoption individually, overlooking the potential synergies and interactions between these two factors. Understanding how access to credit and ICT adoption collectively influence small-scale sugarcane farmers’ income is essential for designing targeted interventions that address the multifaceted challenges faced by farmers in this region. By filling this research gap, this study aims to provide comprehensive insights into the factors shaping farmers’ income dynamics and contribute to evidence-based policymaking aimed at promoting the sustainable development of small-scale agriculture in South Africa.

### 1.1 Theoretical framework

The study is anchored in the Theory of Access, which posits that individuals may possess the right to access a particular resource but may lack the ability to utilize it productively due to structural and relational barriers such as technology, capital, knowledge, authority, labor, social relations, market mechanisms, and identity (Hlatshwayo et al., 2023). Ribot and Peluso (2003), as cited by Mutea et al. (2020), delved into access by examining two variables: the “bundle of rights” and the “bundle of powers”. This perspective emphasizes that access extends beyond mere entitlements to encompass the capability to derive benefits from resources (Ribot and Peluso, 2003). Consequently, the theory suggests that bundles of powers, alongside rights-based access mechanisms, influence how resource users gain control and sustain benefits (Mutea et al., 2020).

In the context of this study, the Theory of Access provides a robust framework for exploring the interplay between credit accessibility, Information Communication Technology (ICT), and the income of small-scale sugarcane farmers. While these farmers have the right to access credit and ICT, their ability to effectively utilize these resources is constrained by various factors. These constraints include a lack of information, suboptimal farming and management practices, inadequate infrastructure, limited agricultural extension services, and restricted access to land. The disconnect between the entitlement to access credit and ICT and the capacity to derive benefits from them hinders small-scale farmers from maximizing their income potential.

Thus, the study aims to assess how determinants of credit accessibility and ICT influence household income among small-scale sugarcane farmers. By applying the Theory of Access, the research seeks to uncover the structural and relational mechanisms that shape farmers’ ability to leverage credit and ICT for income generation. This approach enables a comprehensive examination of the challenges faced by small-scale farmers in fully realizing the benefits of credit and ICT, thereby informing policy interventions and initiatives aimed at enhancing their economic prospects.

## 2 Materials and methods

### 2.1 Description of study area

**Figure 1:**
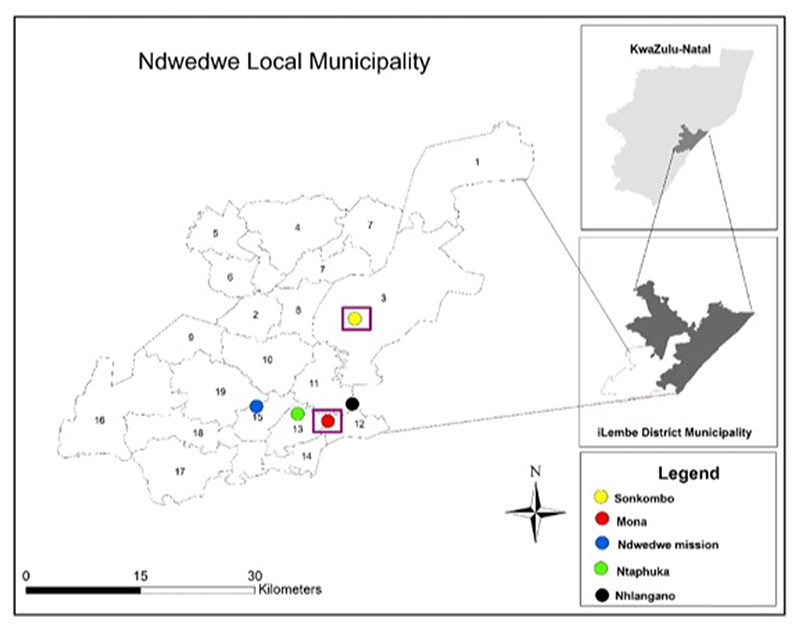
A map showing the location of study sites in Ndwedwe Local Municipality.

The study was carried out in the Ndwedwe Local Municipality of ilembe District Municipality of Kwazulu-Natal. The selected villages are Ndwedwe Mission, Nhlangano and Sonkombo as shown in Figure 1. These villages were selected because they are mostly occupied by small-scale farmers who are involved in the production of sugarcane. These small-scale farmers produce and deliver sugarcane in the same sugar mill (Tongaat-Hulett Sugar-Ltd-Amatikulu Mill). The selected areas also have the same agro-climatic conditions that are suitable to produce sugarcane. Rainfall in these areas is predominantly during the summer months (October-December) while in winter they receive less rain. They are also affected by drought and frost. The annual precipitation ranges from 700 to 1,100 mm (Sibiya and Hurly, 2011). According to the Ndwedwe Local Municipality Integrated Development Plan (IDP), the villages are situated in the south part of Ndwedwe Local Municipality, with an optimum temperature for crop growth of 24–30°C. The mean summer temperature for growth in Ndwedwe Local Municipality is 19°C. Figure 1 shows a map of the location of study sites in Ndwedwe Local Municipality.

### 2.2 Data collection method

The study employed a quantitative approach. A simple random sampling technique was employed to select participants, which is a probability sampling method. According to Tustin et al. (2005) with simple random sampling, the probability of being selected in the sample is known and equal to all members of the population. A questionnaire comprising open-ended and close-ended questions was used as a tool for data collection. The questionnaire explicitly describes the challenges and factors affecting sugarcane productivity by the small-scale sugarcane farmers of Ndwedwe Local Municipality in Kwazulu-Natal. It captured data on the demographic information, socio-economic characteristics of the respondents, as well as institutional and production factors influencing the productivity of the small-scale sugarcane farmers. The questionnaires were translated from English into the local IsiZulu language, the native language in the study area. The university research ethics committee granted ethical clearance approval for this research before it commenced. The research adhered to the policies of the University on research procedures and research ethics.

The questionnaires were administered to respondents through face-to-face interviews. In total, the sample size consisted of 300 small-scale sugarcane farmers (from the three villages (Ndwedwe Mission, Nhlangano, and Sonkombo, respectively). The database of Ndwedwe mission, Nhlangano and Sonkombo has 1,000 small-scale sugarcane farmers, the researcher selected every fifth member as a respondent. Fifty farmers were randomly selected from each of the three selected villages. From a population of 1,000, a minimum ratio of 30% (300 individuals) is advisable to ensure the representativeness of the sample (Neuman, 2007). In as much as this sample size is certainly small to generate robust results, the statistical power of the mathematical model applied in this paper is a reasonably accessible sample in terms of time and costs. There are ~1,000 small-scale sugarcane farmers from each of the three villages that plant sugarcane for marketing and crushing in Ndwedwe Local Municipality. The list of all small-scale sugarcane farmers who delivered sugarcane to the mill at the time of the study is available from the Tongaat-Hulett’s office of Maidstone and the researchers randomly selected every fifth member as a respondent.

### 2.3 Method of data analysis

Following data collection, the data underwent a process of cleaning, recording, and analysis utilizing the Statistical Package for the Social Sciences (SPSS) Version. Descriptive statistics, such as means, standard deviations, frequencies, and percentages, were employed to characterize the socio-demographic attributes of the sampled respondents. Small-scale farmers face a decision regarding the utilization of ICT, a choice that is impacted by various socioeconomic, institutional, and unobservable factors (self-selection). This situation introduces the potential endogeneity issue of the ICT adoption variable during econometric estimation. Previous studies have proposed different approaches to analyzing the impact of a binary endogenous treatment variable (i.e., ICT adoption) on a binary outcome variable (i.e., access to credit), such as the endogenous switching probit (ESP) model (Lokshin and Sajaia, 2011; Nkegbe et al., 2022) and the Recursive Bivariate Probit Regression (RBP) model (Addai et al., 2021; Li et al., 2021). For this study, the RBP model was selected due to its ability to address the endogeneity issue stemming from both observed and unobserved factors. This model also allows for the estimation of a direct marginal effect of ICT adoption on access to credit and vice versa. The Recursive Bivariate Probit Regression (RBP) model estimates two equations (Addai et al., 2021; Li et al., 2021). One equation (Equation 1) focuses on the probability of access to credit, while the other (Equation 2) examines the connection between ICT adoption and the ability of households to access credit.

(1) $$I_{i}^{*}=\eta_{i}X_{i}\xi_{i}IV_{i}+\tau_{i},I_{i}=\{\begin{array}{l} 1,\;if\;I_{i}>0\\ 0,\;otherwise\; \end{array}$$

(2) $$c_{i}^{*}\alpha_{i}I_{i}+\beta_{i}X_{i}+\varepsilon_{i},C_{i}=\{\begin{array}{l} 1,\;if\;C_{i}^{*}>0\\ 0,\;otherwise \end{array}$$

In this context, *I*_*i*_ represents a latent variable signifying the likelihood of a household *I* adopting ICTs, and it is determined by the observed binary variable *I*_*i*_ (where *I*_*i*_ =1 for ICT adopters and *I*_*i*_ =0 for non-adopters). $C_{i}^{*}$ refers to another latent variable representing the inclination toward credit access, determined by the observed binary variable *Ci* (where *Ci* = 1 for credit users and *Ci* = 0 for non-users). *X*_*i*_ is a vector denoting exogenous variables, while IV_*i*_ is an instrumental variable (IV) utilized for RBP model identification. Parameters to be estimated include *η*_*i*_, *ξ*_*i*_, *α*_*i*_, *and* *β*_*i*_, and *τ*_*i*_ and *ε*_*i*_ stand for error terms.

The synthesized valid instrumental variable IV was used in this study as it represents the average number of other ICT adopters (i.e., except for the sampled household) within the same country (Zheng et al., 2021). The synthesized IV is expected to affect a household’s ICT adoption decision, but not affect access to credit directly. Statistically, a Pearson correlation analysis was conducted to test the validity and effectiveness of the IV.

### 2.4 Modeling the joint effects of ICT adoption and access to credit on small-scale sugarcane farmer’s household income

To explore the combined effects of ICT adoption and access to credit on income, the hypothesis was posited that household income could be expressed as a function of ICT adoption, access to credit, and a series of explanatory variables. This can be restated as the regression equation for household income, which is given as follows:

(3) $$Y_{i}=\gamma_{i}I_{i}+\delta_{i}C_{i}+\varphi_{i}X_{i}+\omega_{i}$$

In Equation (3), *Y*_*i*_, represents the dependent variable, which is household income, while *I*_*i*_, *C*_*i*_, and *X*_*i*_ represent independent variables. *γ*_*i*_, *δ*_*i*_, and *ϕ*_*i*_ are the parameters under estimation, and *ω*_*i*_ is an error term. The parameters *γ*_*i*_ and *δ*_*i*_ are indicative of the influences of ICT adoption and access to credit, respectively, on household income. Equation (3) is typically estimated using an ordinary least squares (OLS) regression model. As previously noted, the ICT adoption variable (*I*_*i*_) is endogenous in Equation (3) because farmers self-select themselves as either ICT adopters or non-adopters. Similarly, the access to credit variable (*C*_*i*_) is also potentially endogenous in Equation (3) due to the self-selection issue of becoming credit users or non-users. The endogeneity issue of access to credit variables has been discussed in previous studies (Kumar et al., 2020; Li et al., 2020). Failure to address the endogeneity issues associated with ICT adoption and access to credit variables could lead to biased estimates regarding their joint effects on household income.

In line with previous research (Wooldridge, 2015; Ma et al., 2018), a two-stage selectivity-corrected OLS model was applied to determine the unbiased combined effects of ICT adoption and access to credit on the household income of small-scale farmers. The first stage involves jointly estimating two equations: one for ICT adoption and the other for access to credit. This is accomplished using a seemingly unrelated bivariate probit (SUBP) model, which simultaneously estimates the probability of ICT adoption and access to credit. Unlike in the RBP model estimation, the SUBP model does not include the ICT adoption variable in Equation (2) to prevent a reverse causality relationship between ICT adoption and access to credit.

The SUBP model’s results are utilized to create predicted variables for the endogenous factors. In the second stage, these predicted ICT adoption and access to credit variables, which control for the endogeneity issues, are substituted for the original variables in Equation (4). Consequently, the following selectivity-corrected OLS model can be estimated:

(4) $$Y_{i}=\zeta_{i}I_{i}^{\prime }+\lambda_{i}C_{i}^{\prime }+\varphi_{i}X_{i}+\omega_{i}$$

where *Y*_*i*_ and *X*_*i*_ are variables defined below; *I*_*i*_ and *C*_*i*_ are predicted ICT adoption variable and predicted access to credit variable, respectively; *ζ*_*i*_, *λ*_*i*_, and *ϕ*_*i*_ are parameters to be estimated; *ω*_*i*_ is an error term.

### 2.5 Definition of variables

The income of small-scale farmers is not only influenced by the access to credit and adoption of ICT, however also by several other farm, household, and contextual characteristics. Some of these characteristics may be linked to assess to credit or adoption of ICT, so we need to control for them in the regression models to avoid estimation bias. Age of household head, gender of household head, marital status, educational level of household head, household size, farm and non-farm income, employment status, quantity of own production were identified as socioeconomic and internal factors. These factors mainly affect the production, management, and harvesting systems of farming and the decision of small-scale farmers to be involved in sugarcane production (Hlatshwayo et al., 2022). Access to information, government support, extension support and transportation costs were identified as external variables. These factors affect small-scale farmers’ ability to adopt ICT and be able to access credit. These variables were shown to influence small-scale farmers income in previous studies (Abokyi et al., 2020; Ojo and Baiyegunhi, 2020; Ao et al., 2021; Amrullah et al., 2023). Table 1 gives the definition, variable type and measurements of the independent variables inputted in the regression models.

## 3 Results

### 3.1 Demographic results

Table 2 shows the different sociodemographic factors that affected the adoption of ICT and access to credit by small-scale farmers. The descriptive results showed that about 77% of small-scale farmers had access to credit while 23% did not have access to credit. This means that more farmers were able to use the resources they owned as collateral to acquire loans. The results also revealed that more than 80% of the farmers adopted ICT while 20% did not. This means that most of the farmers were able to adopt information that helped them to boost their production. The current study was dominated by female farmers who amounted to 66% in total, while male farmers were only 34% in total. This is not surprising as smallholder agriculture is mainly dominated by females who provide labor and are mainly involved in the production side. Regarding marital status, the results showed that most (34%) of the farmers were married followed by 25% of farmers who were widowed. Only 6% of the farmers were divorced.

As shown in Table 1 most (37%) of the farmers had primary education while only 14% had tertiary education. This implies that most of the farmers had grade R to grade 7. About 27% of the farmers had no formal education, meaning that they were using their traditional and indigenous knowledge in production. The results showed a high rate (57%) of unemployment percentage among small-scale farmers. This shows that most of the small-scale farmers were unemployed, and they depended more on the production of sugarcane as a source of income. When it comes to non-farm sources of income, more than 41% of the small-scale farmers depended on old pension grants as a main source of income.

### 3.2 Empirical results

### 3.2.1 Factors influencing ICT and access to credit

Table 3 represents the results of the factors influencing the adoption of ICT and access to credit. The results showed that access to credit had a positive and statistically significant at 1% influence on ICT adoption among sugarcane small-scale farmers. Marital status had a negative and significant influence (*p* < 0.1) on ICT adoption. This means that married couples were unable to access ICT. On the other hand, marital status showed a positive and statistically significant (*p* < 0.001) relationship with access to credit. The current results showed that there was a positive and statistically significant relationship between education and ICT adoption among sugarcane small-scale farmers at 5%. Non-farm income had a negative and statistically significant influence on both ICT (at a 10% significant level) and access to credit (at a 5% significant level) among smallholder farmers. Extension support services had a positive and statistically significant (*p* < 0.001) impact on information communication technology of sugarcane small-scale farmers. The age of the household head had a negative and statistically significant (*p* < 0.001) influence on access to credit among small-scale farmers. With regards to gender, the results showed that the gender of the household head had a positive and significant association with access to credit.

### 3.2.2 Joint effects of ICT adoption and access to credit on small-scale farmers income

Table 4 presents the joint effects of ICT adoption and access to credit on small-scale farmers’ income. The variance inflation factor (VIF) was used to control endogeneity and assess how much the variance of an estimated regression coefficient increases when variables are correlated. In this study, all the variables show the mean VIF was 2.617 and it was significant. This means that multicollinearity was not a problem in the regression results. Access to credit and ICT adoption were the variables of interest in this objective, however, they did not show any significant impact on small-scale farmers’ income. Both variables showed a negative sign with no significant influence. This means that the study rejects its hypothesis that access to credit and ICT adoption have an impact on small-scale farmers income.

The results showed that the gender of household head had a positive and statistically significant influence on small-scale farmer’s household income. Marital status showed a positive and statistically significant influence on farmer’s income at a 1% significant level. On the other hand, the study showed that education had a negative and significant relationship with farmer’s income. The results also revealed that employment status and non-farm income had a negative and significant (*p* < 0.001) on small-scale farmer’s income. The current study revealed that extension support and government support had a positive and significant influence on farmer’s income. The study showed surprising results on transport costs. Transportation costs had a positive and significant impact on farmer’s income. The possible explanation is that some of the small-scale farmers had their transport to use during production and they incurred fewer costs or the distance they traveled to suppliers was less and did not cost them so much.

## 4 Discussion

### 4.1 Factors influencing ICT and access to credit

The objective of the study was to assess the factors that affect the adoption of ICT and access to credit and their effect on small-scale farmers’ income. The positive influence of access to credit on ICT adoption among sugarcane small-scale farmers, implies that farmers with access to credit were able to also access information technology and be updated with all the required production information. The results were in line with those of Weng et al. (2023) who found a positive relationship between access to credit and use of the internet. Weng et al. (2023) explained that access to credit and information technology such as the internet can effectively reduce the transaction costs caused by information transmission and search and increase the willingness of farmers to use modern technology. Tchamyou et al. (2019) also found a positive relationship between access to credit and ICT. The authors concluded that access to credit and ICT makes the lives of farmers easy and saves them time.

The negative impact that marital status had on ICT adoption means that married people have a lot of commitment in their personal lives, so they end up being involved less in many social activities. These results are consistent with Sever (2016) who reported that married people with job and household responsibilities experience a lot of pressure which affects their priorities when it comes to communication. Kari (2021) determined the role of marital status in the use of digital library services. The authors found that single women utilized more of the digital library services than married women. They further explained that being married mainly affects decision-making and responsibilities which affect social life. However, marital status showed a positive relationship with access to credit. This implies that married people were able to access credit more than those who were not married. This is because married people have more resources required to access credit than those who are not married. The result is similar to that of Ololade and Olagunju (2013) who found that not being married reduced the probability of having access to credit by 86.3%.

The results indicated that educated farmers were more likely to be able to access and use ICT services. Szymkowiak et al. (2021) outlined that education plays a significant role in advancing the knowledge and skills of an individual. Goldie (2016) also emphasized that education provides scientific and technological advances that improve information and knowledge. The results also revealed that there was a negative and statistically significant impact (at 1% significant level) of level of education on access to credit. This means that farmers who are educated are using other forms of income to finance their agricultural production. On the contrary, Kiplimo et al. (2015) found that educational level had a positive effect on access to credit financial services. Haryanto et al. (2023) also reported that farmers with higher levels of education have more advantages to have secured collateral which enables them to access credit. Hussein (2007) also found contradicting results with the current study and reported that educated farmers can access and understand information on credit terms and conditions which allow them to complete their application forms correctly.

The negative impact of non-farm income on ICT adoption and access to credit means that farmers who were relying on non-farm income were not able to access both ICT and credit. The results were contrary to those of Kiplimo et al. (2015) who found that farmers who were employed outside the farm were able to access credit. The authors explained that farmers were able to generate more income outside the farm and accumulate more assets that would be used as collateral when seeking credit services. On the other hand, extension support services had a positive impact, implying that small-scale farmers who were getting support from extension agencies were more likely to access more information on their production. The results concur with that of Wossen et al. (2017) who found a positive relationship between extension services and access to ICT. The study recommended that there is a need for extension services to help farmers in the expansion of financial markets that help in improving farmers “welfare and efficiency”.

The positive relationship between age and access to credit implies that as the age of small-scale farmers increases, access to credit decreases. The current results are consistent with Chandio et al. (2017) who found that the age of households had a negative and significant effect on farmers’ access to credit. The study therefore concluded that there is a need for institutional sources of credit to improve their loaning schemes to better suit the diversified needs of small farmers. In contrast, Kehinde and Ogundeji (2022) found that age was among the socio-demographic factors that positively influenced the productivity of those farmers who have access to credit. The authors reported that as farmers get old, they acquire enough assets that will serve as collateral and be able to secure credit. The positive relationship between gender and access to credit means that women had the same access to credit as men do. This also meant that women were able to acquire sufficient credit that helped them to finance their production. The results concur with the results of Chandio et al. (2017) who found a positive relationship between gender and access to credit. Kehinde and Ogundeji (2022) found different results which showed that male farmers had better access to credit than their female counterparts. The authors further explained that females are not generally involved in decision making they are more involved in in-house chores that hinder them from participating in many things.

### 4.2 Joint effects of ICT adoption and access to credit on small-scale farmers income

The positive relationship between gender and small-scale farmers income means that as females were also able to utilize all the resources, they had in farming and be able to generate more income. The results are similar to that of Abokyi et al. (2020) who also found a positive relationship between gender and income. In the contrary, Tolno et al. (2015) found that gender was significant and negatively related to farmer’s income. In the same vein, Abokyi et al. (2020) found that male-headed households had better farm income when compared to female-headed. The authors then explained that their findings are an important indicator of household decision-making whereby in a traditional setup, key decisions in a household are made by men. The results also showed that married farmers improved their income generation. This means that as more farmers get married, they join their resources for production and generate more farm income. These results were opposed to those of Ojo and Baiyegunhi (2020) who found that marital status was statistically significant and negatively related to farmers’ net income. The authors explained that married households have large family sizes which makes them use their net income in other activities than production. The negative impact that education had on farm income means that as farmers get more educated their level of participation in farm decreases which decreases their income. The plausible explanation is that educated people opt for other jobs that pay better than being involved in farming full-time. However, Serin et al. (2009) found that formal education and practical education in the form of utilizing expert consulting and training services also increase the productivity and income of farmers. Ao et al. (2021) also found that formal education was among the sociodemographic factors that had a significant positive impact on farming income levels. The study outlined that it is important to improve and strengthen agricultural technical training of farmers and increase their level of education.

Employment status and non-farm income had e detrimental effect on income meaning that farmers who were employed outside the farm were not using their off-farm income to finance their agricultural production and generate more farm income. In line with these findings, Odoh et al. (2020) found a lower level of income generation from farm activities than non-farm activities. Odoh and Nwibo (2016) also found that in Nigeria non-farm sector is a major contributor to employment and income generation of rural households, contributing up to 63% of household income.

Agricultural extension services play a crucial role in boosting small-scale sugarcane productivity which in turn improve farm income and increase food security and rural livelihoods. These services are provided by the government as an intervention to support small-scale farming. In this study the agricultural showed a positive influence, the imply that small-scale farmers were receiving adequate support from extension services which increased their productivity and income. The results of the current study are like those of Baiyegunhi et al. (2019), who found that government extension programs significantly contributed to an increase in the net farm income of the participants. Amrullah et al. (2023) also reported that access to extension services plays an important role in agricultural income. The study suggested that the government needs to increase public investment in extension as it optimizes the potential impact on technology adoption and agricultural income, which also affects the distribution of the welfare of rural smallholder farmers.

## 5 Conclusion and policy recommendations

The sugarcane sector plays a vital role in South Africa’s GDP, contributing significantly to socioeconomic development by fostering job creation, resource organization, income generation, and the development of transport and communication networks. In assessing the factors influencing the adoption of ICT and access to credit among small-scale farmers, this study identified key socio-demographic factors impacting technology adoption and credit access. It was observed that factors such as access to credit, education, and extension support positively influenced the adoption of ICT, while marital status and non-farm income had adverse effects. Similarly, gender and marital status positively contributed to access to credit, while age, education, and non-farm income exhibited negative relationships. Despite the positive influence of these factors on technology adoption and credit access, the study did not find significant impacts on small-scale farmers’ income. This challenges the hypothesis positing a direct correlation between access to credit, ICT adoption, and farm income. Nevertheless, several socio-demographic factors, including gender, marital status, extension support, government assistance, and transportation costs, were found to positively impact farmer income, while education, employment status, and non-farm income had negative effects.

To enhance small-scale farmers’ income and promote sustainable agricultural development, it is imperative to implement targeted policy interventions. First and foremost, efforts should focus on improving access to credit and fostering ICT adoption among farmers. This can be achieved through educational programs, workshops, and training initiatives aimed at equipping farmers with the necessary skills and knowledge to navigate formal credit processes and adopt modern information technologies effectively. Furthermore, extension workers should play a proactive role in providing advisory support to small-scale farmers seeking agricultural credit. Creating a conducive investment environment and implementing government policies to lower loan interest rates and collateral requirements are essential steps in facilitating credit access for farmers. Additionally, ongoing support from extension services is crucial for enhancing farmers’ productivity and income levels. By addressing the identified socio-demographic factors and implementing targeted policy interventions, stakeholders can foster an enabling environment for small-scale farmers to thrive, ultimately contributing to the sustainable development of the sugarcane sector and the broader agricultural landscape in South Africa.

### 5.1 Limitations and directions for future research

The study used only one province (KwaZulu Natal) and has been limited to only one local municipality that is Ndwedwe Local Municipality. Future studies can perform the same research across all nine provinces of South Africa where small-scale sugarcane farmers are located. The findings can be used to compare small-scale sugarcane farmers’ challenges and opportunities in their different working environments. Additionally, the study focused on only one agricultural community produced by small-scale farmers. Future research may explore the impact of access to credit and adoption of ICT across all the various agricultural commodities produced by small-scale farmers. The findings will also help develop a comprehensive report that will be submitted to policymakers, government, and other stakeholders for necessary interventions.

## Figures and Tables

**Table 1:** The explanatory variable that affect adoption of ICT and access to credit among sugarcane small-scale farmers.

Variable name	Variable type and measurement	Rationale
Age of the household head	Participant’s age in years	Age can influence access to credit and ICT adoption, as older farmers may face different barriers and have different preferences compared to younger farmers (Kiplimo et al., 2015). Older farmers may have accumulated more experience and assets, potentially improving their creditworthiness and ability to adopt ICT. However, older farmers may also face technological barriers or financial constraints that hinder their adoption of ICT (Chandio et al., 2017).
Gender of household head	If the respondent is male, 1; otherwise, 0	Gender may impact access to credit and ICT adoption due to potential disparities in resources, opportunities, and societal norms (Abdallah, 2016). Gender equity initiatives have been shown to improve access to credit for female farmers, reducing gender disparities (Chandio et al., 2017). Additionally, societal norms and biases may influence women’s access to and utilization of ICT (Abokyi et al., 2020).
Marital status	If the participant is married, 1 is assigned; otherwise, 0	Marital status can affect access to credit and ICT adoption, as married individuals may have different financial responsibilities and time constraints (Odoh et al., 2020). Married farmers may benefit from shared resources and stability, potentially improving their access to credit. However, marital responsibilities may also limit the time available for engaging with ICT (Sever, 2016).
Education	Years of education (continuous)	Education level can influence access to credit and ICT adoption, as higher education may correlate with better understanding and utilization of technology and financial services (Kiplimo et al., 2015). Farmers with higher levels of education are more likely to adopt ICT and access credit due to their enhanced understanding of modern agricultural practices and financial systems.
Employment status	If the respondent is employed, 1; otherwise, 0	Employment status may impact access to credit and ICT adoption, as employed individuals may have more stable income and access to financial resources (Abdallah, 2016). Employed farmers may have greater financial capacity to invest in technology and qualify for credit.
Nonfarm income	If there is a person who works for income, 1; otherwise, 0	Non-farm income may affect access to credit and ICT adoption, as additional income sources may influence farmers’ financial capacity and willingness to invest in technology (Abdallah, 2016). Farmers with non-farm income may have higher disposable income and be more willing to invest in ICT and qualify for credit.
Extension support	If the participant had access to extension support, the answer was 1, otherwise it was 0.	Extension support can facilitate access to credit and ICT adoption by providing farmers with necessary information, training, and resources (Baiyegunhi et al., 2019). Farmers who receive extension support are more likely to adopt modern farming practices and technologies, improving their creditworthiness and ability to utilize ICT.
Government support	If the participant had access to government support, the answer was 1, otherwise it was 0.	Government support programs can impact access to credit and ICT adoption by providing financial assistance, infrastructure, or policy incentives (Baiyegunhi et al., 2019). Government-supported credit programs and ICT initiatives can expand farmers’ access to financial services and technology, promoting agricultural development.
Seed cane age	Seed cane age (continuously)	Seed cane age may indirectly influence access to credit and ICT adoption by affecting sugarcane productivity and farmer income (Thibane et al., 2023). Younger seed cane may lead to higher productivity and income, potentially improving farmers’ creditworthiness and ability to adopt ICT. Older seed cane may hinder production efficiency, impacting farmers’ financial capacity and willingness to invest in technology.

**Table 2:** Socio-demographic factors of small-scale sugarcane farmers in Ndwedwe Local Municipality.

Variable	Percentage (%)
Access to credit	
Yes	77
No	23
Adoption of ICT	
Yes	80
No	20
Gender	
Male	34
Female	66
Marital status	
Single	21
Married	34
Widowed	25
Divorced	6
Living with partner	14
Level of Education	
No formal education	27
Primary school level	37
Secondary level	22
Tertiary level	14
Employment status	
Unemployed	57
Employed temporal	33
Employed permanent	10
Non-farm income	
Salaries	2
Old age pension	41
Disability grant	4
Child support grant	28
Foster care	12
Business	13

**Table 3:** Factors that influence ICT and access to credit-recursive bivariate probit regression model.

Variable	Coefficient	Sth. err.	*P*-value	Coefficient	Sth. err.	*P*-value
	Information communication technology			Access to credit		
Access to credit	1.591	0.293	0.000***			
Age	0.006	0.015	0.675	−0.043	0.009	0.000***
Gender	0.393	0.321	0.220	0.828	0.197	0.000***
Marital status	−0.182	0.104	0.080*	0.280	0.068	0.000***
Education	0.380	0.183	0.038**	−0.501	0.097	0.000***
Employ status	−0.087	0.250	0.729	−0.016	0.143	0.913
Non-farm income	−0.205	0.113	0.069*	−0.174	0.082	0.033**
Extension support	1.003	0.277	0.000***	0.168	0.174	0.335
Government support	0.137	0.173	0.427	0.164	0.108	0.128
Seed cane age	0.107	0.160	0.504	0.031	0.093	0.741
_cons ICT	−1.583	1.528	0.300			
_cons access to credit				2.077	0.837	0.013
/atanrho				−10.825	262.891	0.967
Rho				−1		−1

***, **, * indicate significance at 1, 5, and 10% level, respectively.

**Table 4:** The effect of ICT and access to credit on sugarcane small-scale farmer’s income- selectivity-corrected ordinary least square regression model.

Variable	Coefficient	Sth. err.	*P*-value	VIF
e2				2.617
Gender	12.380	0.903	0.000***	1.371
Marital status	5.336	0.321	0.000***	1.29
Education	−8.493	0.542	0.000***	2.117
Employment status	−2.945	0.590	0.000***	1.156
Non-farm income	−3.699	0.282	0.000***	1.498
Access credit	−0.221	1.089	0.840	1.738
ICT	−0.345	1.768	0.768	1.765
Extension support	3.459	0.740	0.000***	1.031
Government support	2.322	0.477	0.000***	1.118
Seed cane age	1.023	0.388	0.789	1.106
Low-income	0.290	1.051	0.783	1.727
Transport costs	0.000	0.000	0.018**	1.107
Mean VIF				1.49
e2 (residual ICT_access to credit)	−56.985	2.736	0.000***	
Constant	86.594	3.106	0.000	
Mean dependent var	55.897	SD dependent var	15.646	
*R*-squared	0.843	Number of obs	300.000	
*F*-test	128.487	Prob > F	0.000	
Akaike crit. (AIC)	1,970.917	Bayesian crit. (BIC)	2,019.066	

***, ** indicate significance at 5 and 10% level, respectively.

## Data Availability

The raw data supporting the conclusions of this article will be made available by the authors, without undue reservation.
